# Responses to Pheromones in a Complex Odor World: Sensory Processing and Behavior

**DOI:** 10.3390/insects5020399

**Published:** 2014-06-17

**Authors:** Nina Deisig, Fabienne Dupuy, Sylvia Anton, Michel Renou

**Affiliations:** 1 Institut d’Ecologie et des Sciences de l’Environnement de Paris (iEES-Paris), UMR 1392, Département d’Ecologie Sensorielle, INRA, Route de Saint-Cyr, 78026 Versailles Cedex, France; E-Mails: nina.deisig@versailles.inra.fr (N.D.); michel.renou@versailles.inra.fr (M.R.); 2 Laboratoire RCIM, Université d’Angers, UPRES-EA 2647, USC INRA 1330, SFR 4207 QUASAV, UFR Sciences, 2 Boulevard Lavoisier, 49045 Angers, France; E-Mail: fabienne.dupuy@angers.inra.fr

**Keywords:** sex pheromone, plant odor, odor interactions, neural mechanisms, mixture processing, orientation behavior

## Abstract

Insects communicating with pheromones, be it sex- or aggregation pheromones, are confronted with an olfactory environment rich in a diversity of volatile organic compounds of which plants are the main releaser. Certain of these volatiles can represent behaviorally relevant information, such as indications about host- or non-host plants; others will provide essentially a rich odor background out of which the behaviorally relevant information needs to be extracted. In an attempt to disentangle mechanisms of pheromone communication in a rich olfactory environment, which might underlie interactions between intraspecific signals and a background, we will summarize recent literature on pheromone/plant volatile interactions. Starting from molecular mechanisms, describing the peripheral detection and central nervous integration of pheromone-plant volatile mixtures, we will end with behavioral output in response to such mixtures and its plasticity.

## 1. Introduction

Insects often use pheromones to communicate intraspecifically and resource-related volatiles to forage or localize hosts. These two functional classes of semiochemicals are generally involved in different types of behaviors for instance sexual or social for pheromones and feeding or reproductive behaviors for plant volatiles but may interact with each other. Pheromones in general and more specifically sex pheromones are mostly blends of a few components emitted in a species-specific ratio. In moths, which will be in the focus of the present review, males have a highly sophisticated detection system to recognize very small amounts of the female-emitted sex pheromone and orient towards a small source based on spatio-temporal distribution of pheromone filaments [1]. On the other hand, plants release a huge diversity of compounds and often in large amounts. Some of these volatiles, frequently assembled in complex mixtures of many compounds, indicate important resources for herbivorous insects such as feeding or oviposition sites. In turn, herbivory-induced volatiles might repel herbivorous insects [2] while attracting their parasitoids [3] and non-host plants repel specialized herbivorous insects [4]. Many other plant volatiles seem to be neutral as far as the absence of characterized responses may allow to conclude.

**Figure 1:**
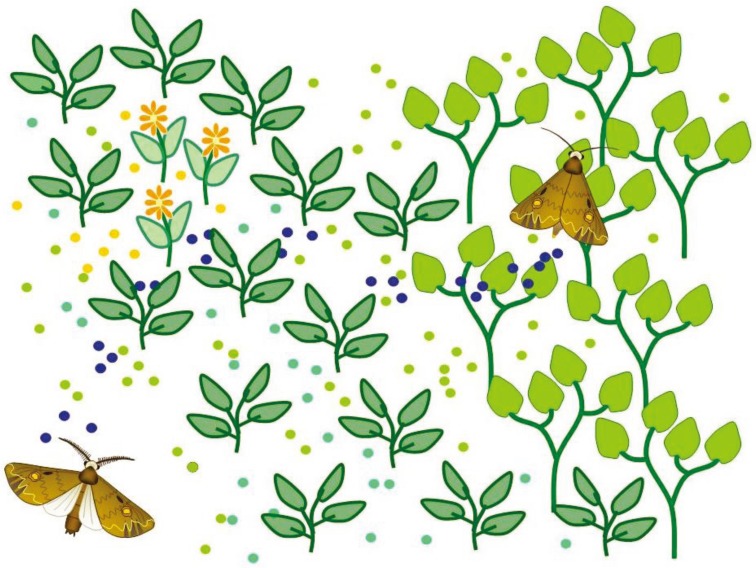
Orientation of male moths towards the female-emitted sex pheromone in a natural environment. The pheromone (blue) is emitted in small amounts from a point source, an isolated female, whereas flower and green leaf volatiles (yellow and green) from host and non-host plants are highly variable and are often emitted in large amounts and from multiple sources. Detailed physical models for such complex signal combinations are missing but we can extrapolate that they result in a meandering but relatively narrow pheromone plume in a more dispersed, but still heterogeneous cloud of diverse plant volatiles. More homogenous clouds are probably found in highly simplified agro-ecosystems where plants of the same species, variety, and growing stage dominate over large surfaces.

One of the major questions arising due to the co-existence of these two functional odor classes is whether interactions occur between pheromones and plant volatiles in the insect’s perceptual and processing system. Due to their different temporal occurrence in nature, we further discuss if a specific long-lasting background of plant odors, such as for example host plant cues, might facilitate orientation of a male moth following a female sex pheromone plume in which it encounters pulses of the species-specific sex pheromone. Indeed in several species, females release their pheromone from specific host plants and the host volatile emissions stimulate pheromone emission (reviewed in [5]). On the other hand, large amounts of plant odors might represent a non-specific and highly variable odor landscape when a male insect is trying to orient towards small amounts of sex pheromones. In natural conditions, pheromone and plant volatiles, which are released from spatially distinct sources, probably do not reach the olfactory organs in synchrony, as a one-source odor mixture would do. Furthermore, due to air turbulences, odor plumes emanating from small sources, such as one female emitting sex pheromone or a single flower, are fragmented and result in a highly intermittent signal at a distance from the source [6]. Although the blends of volatile compounds that are released by one plant may result in odor plumes very similar to that of pheromones [7], in a natural environment many plant sources release odors simultaneously. Compared to the minute pheromone source, the numerous inflorescences of trees, or the huge number of individual plants in a field, probably behave as a very large source, with a quite different downwind distribution of odors. It might thus be a challenge for males to extract the quality, intensity, and temporality of the pheromone signal from a complex plant odor environment which risks masking or altering the behaviorally relevant information (Figure 1).

In this article we review the literature on pheromone-plant odor interactions at different integration levels from signal reception up to protocerebral integration, with an emphasis on mechanistic aspects. We then analyze the plasticity of such interactions as a function of intrinsic and extrinsic factors and describe what is known on the orientation behavior towards mixtures of pheromone and plant odors. Finally, we briefly discuss what is known about consequences of pheromone-plant odor interactions at an evolutionary scale.

## 2. The Reception of Sex Pheromones and Plant Volatiles

In insects, olfactory signals are detected by olfactory receptor neurons (ORNs) housed in the cuticular sensilla mainly situated on the antennae. Hydrophobic odor molecules penetrate the cuticle through wall pores and are transported by odorant binding proteins through the aqueous sensillar lymph surrounding ORN dendrites. Olfactory receptors situated within the dendritic membrane bind odor molecules and lead to transduction of the chemical signal into a receptor potential which is then transformed into action potentials and transmitted along the ORN axon to the brain. The antennal nerve comprising all ORN axons enters the antennal lobe (AL) of the brain, where primary processing of the incoming information is happening. This integrated information is then transmitted to higher olfactory brain centers, *i.e.*, the lateral protocerebrum and the mushroom bodies [8].

The components of female moth pheromone blends are detected by specialized olfactory receptor neurons (Phe-ORNs) housed in long trichoid hairs on the male antennae. In many cases, each Phe-ORN is narrowly tuned to one of the components of the pheromone blend. Male antennae also contain general odorant receptor neurons (GO-ORNs) showing various degrees of chemical specificity. While many ORNs responding to general odors are broadly tuned, also some ORNs with high specificity for individual plant volatiles have been described [9,10]. GO-ORNs are housed in a variety of morphological types of sensilla, including olfactory hairs and non-hair types of sensilla. So far, Phe-ORNs and GO-ORNs have been found in separate sensilla with the exception of a few sensilla auricillica in *Cydia pomonella*, which house both types of neurons [11]. Thus, the general picture for moths has so far been a functional and anatomical separation of two sub-systems of odor detection, one for the sex pheromone, the other for general odorants. However, chemical tuning is not exclusive and Phe-ORNs can also, in few cases, respond to plant odorants.

**Figure 2:**
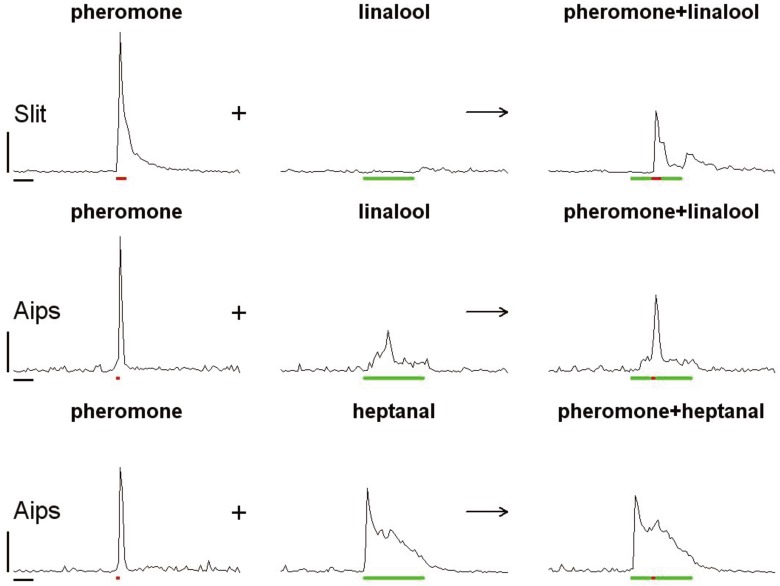
A background of a plant volatile affects the intensity and the dynamics of the responses to a pheromone pulse by specialist olfactory receptor neurons in moth antennae. Instantaneous spike frequencies calculated as the slope of a cumulative function [21] are shown over time for typical Phe-ORNs. In *Spodoptera littoralis* (Slit, **upper row**) pheromone specialized neurones respond to a short pulse of the main pheromone component (Z9,E11-14:Ac) by a step increase in their firing response (**left**); a prolonged stimulation with linalool does not increase spontaneous firing (**middle**); simultaneous presentation of linalool and pheromone results in reduced peak firing frequency, narrowing of the response and a post-background rebound. While linalool acts as an antagonist of pheromone reception in *S. littoralis*, it is a weak agonist in *Agrotis ipsilon* leading to weak activation as single stimulus and mixture suppression together with pheromone (Aips, middle row). The component of linden flower aroma, heptanal, strongly stimulates Pher-ORNs (agonism, Aips, lower row) in *A. ipsilon* but masks the response to a pheromone pulse. Red bars = pheromone stimulation; green bars = volatile plant compound stimulation. Graphs present the frequency curves averaged on responses recorded from 10 to 20 different sensilla. Horizontal scale bar 1 s, vertical scale bar 50 spikes/s. Data from [13,16].

Although Phe-ORNs generally do not respond to plant volatiles, cases of interactions between plant odor and pheromone reception have been known for a long time. For instance, in *Yponomeuta* ssp*.* moths when the pheromone component *cis*-11-tetradecenyl acetate and the host-plant volatile geraniol were applied simultaneously, geraniol inhibited the responses to the sex attractant [12]. When adding the plant volatile linalool to the pheromone, a suppressive effect was observed at the level of the pheromone-specific ORNs in *Spodoptera littoralis* (Figure 2), which improved the temporal resolution of pheromone pulses [13]. However, not all plant volatiles interfere with pheromones in the same way, and a very abundant plant compound like isoprene did not affect pheromone detection in the same species [14]. Also in *Agrotis ipsilon*, the flower volatile heptanal, reduced the response of Phe-ORNs to the pheromone blend when applied simultaneously, but it stimulated the firing of Phe-ORNs when used as a single stimulus, acting as a partial agonist [15,16] (Figure 2). In turn, linalool and (Z)-3-hexenol (Z3-6:OH), but not β-ocimene, when presented together with (Z)-11-hexadecenal (Z11-16:Ald), increased the response of the Phe-ORNs in a synergistic way in the noctuid moth *Helicoverpa zea* [17]. Another case of synergy in moths has been observed in *Heliothis virescens*, following stimulation with β-caryophyllene plus the pheromone component Z11-16:Ald [18]. However, the same study reported decreased firing responses of Phe-ORNs to their specific pheromone component when either one of five other plant compounds or another pheromone component was added. Thus, cases of synergy remain exceptional, with most studies reporting mixture suppression ([13] and references therein).

Evidence for interactions at the pheromone receptor sites has been recently found in *Heliothis virescens* [19]. Several plant volatile compounds, such as linalool, linalyl-acetate, Z3-6:OH, and geraniol, but not isoamyl-acetate reduced the pheromone-evoked calcium release in the areas of the primary olfactory center receiving Phe-ORN projections. As calcium sensitive dyes applied in a bath essentially reveal the activity of ORN axons, which represent the majority of synaptic sites within the AL as compared to central neurons [20], this suggests a reduction of pheromone input. Competitive fluorescence binding assays with the *H. virescens* pheromone binding protein HvirPBP2 purified after bacterial expression showed that plant volatiles did not interfere with the binding of Z11-16:Ald to its pheromone binding protein. In turn, linalool reduced the responses to Z11-16:Ald of a stable cell line expressing the pheromone receptor HR13, evidencing a direct effect already at the receptor level.

Since general odorants can interfere with the binding of pheromone to its receptors, their accumulation in the pheromone sensilla might be detrimental to signal detection. Pheromone molecules are rapidly inactivated by enzymatic degradation [22] in the olfactory tissues by pheromone degradation enzymes (PDEs) expressed in pheromone sensilla. The PDEs are postulated to contribute to signal termination and maintenance of the capacity of the neurons to follow fast temporal changes in the signal. Degradation of the great diversity of volatile molecules, including compounds potentially harmful to neurons, can be achieved by olfactory degrading enzymes (ODEs) with broader substrate spectra. A high diversity of cytochrome P450 is expressed within moth antennae [23], showing that the antennae are a key site for the degradation of a broad range of exogenous molecules. Alternatively, PDEs might have larger substrate spectra than expected. For example, pheromone components and plant components have been shown to be degraded by the same carboxyl esterase in *S. littoralis* [24].

With respect to the need for a highly sensitive extraction of the pheromone signal, the more frequently found inhibition of the pheromone detection system by plant odorants appears counter-intuitive at first. However, the same linalool background improved temporal resolution of pulsed pheromone signals in Phe-ORN and stimulated some of the general odorant ORNs [13]. Thus, it is important to consider how the different levels of the olfactory system will process this input to be able to evaluate the consequences on odor driven behavior.

## 3. Pheromone-Plant Odor Interactions and Signal Coding

**Figure 3:**
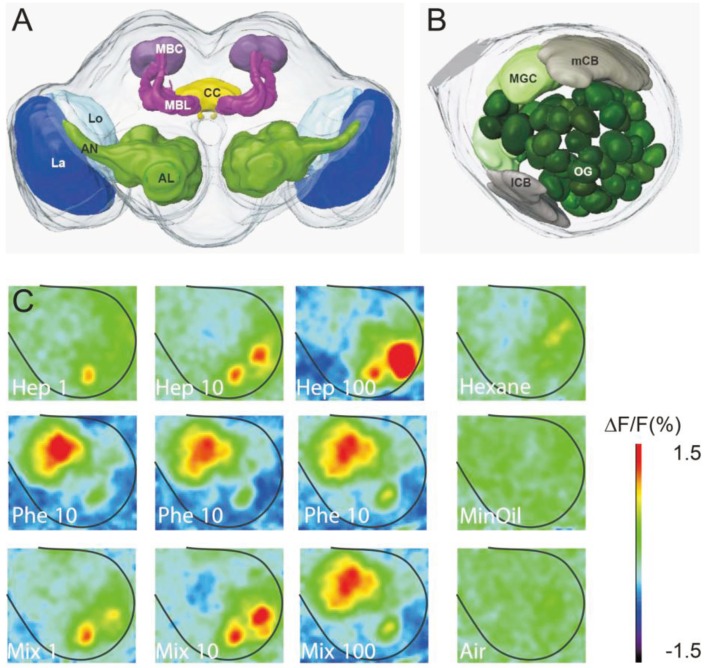
The *Agrotis ipsilon* brain and pheromone-plant odor interactions visualized in the antennal lobe by *in vivo* calcium imaging. (**A**) Schematic representation of the noctuid moth brain and (**B**) the antennal lobe. (**C**) False color-coded images of optically recorded antennal lobe activity. In response to different doses of heptanal (Hep 1, 10 and 100 μg), the behaviorally active sex pheromone blend (Phe) at 10 ng and the heptanal-pheromone blend mixture (Mix, containing 10 ng of pheromone blend and 1, 10 or 100 μg of heptanal). Hexane, the solvent of the pheromone, mineral oil (MinOil), the solvent of heptanal, as well as air, are control stimulations. All maps are scaled to the same minimum/maximum as indicated by the color scale and originate from a single five days-old male moth. AL antennal lobe, AN antennal nerve, CC central complex, La lamina, lCB lateral cell body cluster, Lo lobula, MBC mushroom body calyces, MBL mushroom body lobes, mCB medial cell body cluster, MGC macroglomerular complex, OG ordinary glomeruli.

Olfactory information is transmitted via the axons of ORNs to the primary olfactory center, the AL, which forms part of the deutocerebrum of the insect brain (Figure 3a). Each AL is composed of spherical functional subunits, the olfactory glomeruli (e.g., [25]). All ORN axons converging onto the same glomerulus express the same olfactory receptor (OR), and this glomerulus thus receives relatively specific olfactory information, depending on the specificity of the corresponding OR [26,27]. Depending on the affinities of ORs to a ligand, each odorant elicits activity in an odor-specific ensemble of glomeruli [28]. In insects using sex pheromones, such as moths, a few large glomeruli receiving axons from Phe-ORNs form the macroglomerular complex (MGC) in males, whereas ordinary glomeruli (OG), receiving information about general odors are in most cases sexually isomorphic (e.g., [29]) (Figure 3b). However, cases of sexually dimorphic OG with enlarged glomeruli in females have also been described [30,31]. A special feature of the pheromonal system resides in the high specificity of the ORs for their ligand [32]. Each pheromone component is detected by a distinct functional ORN type [33,34], which projects into a unique glomerulus of the MGC ([35,36] and references therein). Moreover, a large number of ORNs dedicated to the detection of sex pheromone components in male moths converges onto only a few output neurons (projection neurons, PNs) arborizing in the MGC, allowing high sensitivity and a large dynamic range [29,37,38,39]. The olfactory pathway in male moths is thus divided into a pheromone-specific system and a general odor system. Within glomeruli, ORNs form synapses with dendritic arborizations of local neurons (LNs) and PNs. LNs are restricted to the AL and interconnect different glomeruli. A large proportion of LNs is GABAergic, forming an inhibitory network within the AL, however, some excitatory LNs have been described [40,41,42]. LNs play a role in the modulation of olfactory responses through peptide action [43,44]. PNs have dendritic arborizations within the AL and transmit olfactory information to higher-order brain centers, such as the mushroom bodies (MBs) and the lateral protocerebrum (LP) (for review see [25]) (Figure 3a).

In processing odor mixtures, animals can perceive either the elements of a stimulus mixture (elemental processing, e.g., [45]) or treat a mixture as entity different from its elements (synthetic processing, e.g., [46]). Odor mixture processing within the AL has been studied extensively for blends of different pheromone components in moths and for blends of plant-related odors in the honey bee and the fruit fly. Component-specific PNs, PNs and LNs responding to several components and blend-specific PNs and LNs have been described for the MGC in several moth species ([47] and references therein). Thus there is evidence for both elemental and configural coding of sex pheromone blends at the level of the antennal lobe. While AL input of plant odor mixtures in honey bees has been shown to be essentially elemental, different tuning and response patterns of different functional classes of plant odor-responding ORNs in different insects indicate a more important role of across fiber processing for plant odor mixtures [48,49]. Calcium signals measured by *in vivo* calcium imaging in response to plant odor mixtures included the glomeruli responding to the single elements in the honey bee [50]. It has further been shown in the honey bee that similarity between a mixture and its components can be linearly predicted based solely on the magnitude of the responses to each component (*i.e.*, number of glomeruli), despite the occurrence of overall inhibitory phenomena at the AL input level [51]. Such elemental mixture processing at the AL input level has also been found in moths [52] and *Drosophila* [53]. In contrast, for the AL output level, strong interactions have been found between component signals within the AL networks (*Drosophila:* [53], honey bees [54]), assigning unique properties to each mixture’s representation. Thus, PN mixture representation, making similarity relationships between mixture and components less predictable based on component information, *i.e.*, less elemental, is the result of subtle reformatting within the AL, generated by lateral inhibition involving LNs [53,54].

Much less information is available about AL processing of mixtures of pheromone and plant odors. So far, coding of plant odor-pheromone mixtures in the AL has been studied in several Lepidoptera species belonging to different families with no close phylogenetic relationships. Some studies have been conducted on both subsystems *i.e.*, the OG and the MGC, at the AL input as well as at the output level, and for different mating statuses. The first important finding is that neurons within both subsystems do not respond as specifically as previously postulated. Indeed many OG neurons responding to a specific plant odor also respond to the sex pheromone, and many pheromone sensitive neurons of the MGC also respond to plant odors, probably due to primary integration within the AL [55,56,57,58]. In some species, however, such as *A. ipsilon*, pheromone-specific ORNs may also respond to certain plant odors, such as heptanal at high doses [16], and in this case we cannot conclude if plant odor responses in central neurons are due to input from the periphery or to primary processing within the AL.

**Figure 4:**
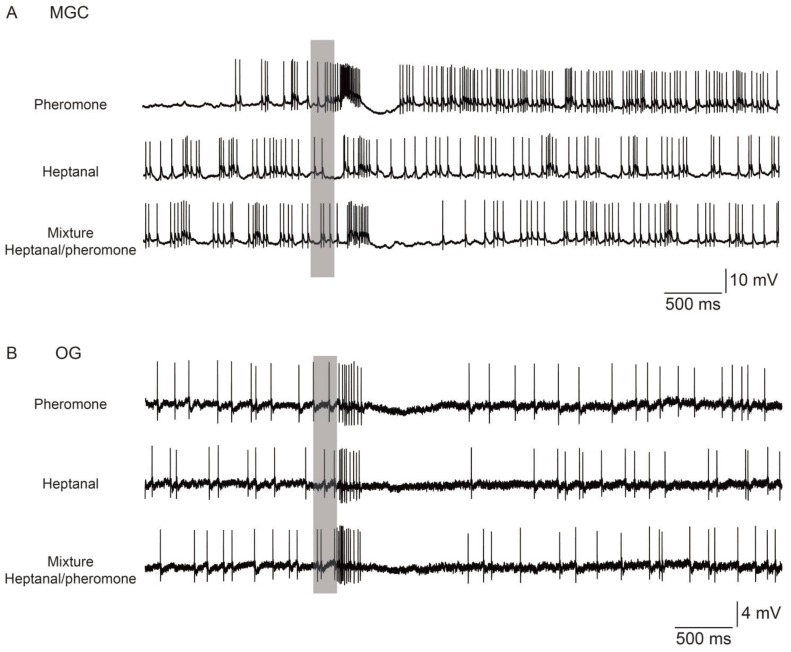
Interactions between pheromone and heptanal induce opposite effects in the response of neurons of the two sub-systems of the antennal lobe in *Agrotis ipsilon*. The different traces show examples of antennal lobe neuron responses to three olfactory stimulations (grey bar) (*i.e.*, 1 ng of the sex pheromone, 100 μg of heptanal and the mixture of pheromone and heptanal) recorded intracellularly. (**A**) In the pheromonal sub-system (MGC), the neuron shown responds to the pheromone (blend) with a strong excitation followed by an inhibition phase (upper trace) and no or a weak response to heptanal. The presentation of heptanal together with the pheromone induces a reduced response, showing a suppressive effect (lower trace). (**B**) In the non-pheromonal sub-system (OG), neuronal responses are stronger for stimulation with heptanal (middle trace) than with pheromone (blend, upper trace). The responses to heptanal are further enhanced when adding the pheromone showing an additive mixture effect (lower trace).

When investigating pheromone-plant odor interactions within the pheromonal sub-system (MGC) of virgin *A. ipsilon* males, a suppressive effect of the presence of plant odor is observed on pheromonal responses at different levels. MGC-PN responses to sex pheromone decrease in the presence of the floral odor heptanal (*i.e.*, neurons respond with longer latencies, lower spiking frequencies, and shorter excitatory phases) [15,56] (Figure 4a). Recordings of the AL input obtained using calcium imaging show lower response intensity to the pheromone-heptanal mixture than to the pheromone alone [15] (Figure 3c). However, a similar suppressive effect was already observed at the level of the pheromone-specific ORNs (*cf.* part 2). These results indicate that the suppressive effects might originate mainly from mixture interactions at the peripheral level. Suppressive responses to the plant odor-pheromone mixture in both pheromone sensitive ORNs and PNs lead to improved resolution of pulsed stimulation [13,56]. The opposite effect was observed in two other moth species, the silk moth *Bombyx mori* and the codling moth *Cydia pomonella*. Adding *cis*-3-hexen-1-ol, a volatile component emitted from the host plant, mulberry, to the pheromone compound bombykol, enhanced the response to the pheromone in intracellularly recorded PNs innervating the MGC (*i.e.*, neurons exhibited higher firing rates) in *Bombyx mori* [59]. Increased responses were also observed in PNs innervating the cumulus of the MGC in *C. pomonella* when stimulated with the main pheromone component codlemone in a blend with acetic acid [57].

In the non-pheromonal sub-system (OG), interactions between pheromone and plant odors are more complex, as the effects are not only different depending on the species investigated, but also depending on the neuron type investigated and the physiological state of the animal, *i.e.*, the mating status. Indeed, synergistic effects of the pheromone on heptanal responses were observed in about half of the OG-PNs recorded in virgin *A. ipsilon* males that did not respond to pheromone alone [60] (Figure 4b). This result was partly confirmed by further recordings that showed responses to pheromone in OG-PNs and LNs and additivity *i.e.*, higher maximum firing frequency, shorter response latencies, and longer excitatory phases were observed in response to the mixture of heptanal and sex pheromone compared to heptanal alone [58]. Interestingly, these additive effects increase the resolution of pulsed stimuli for the mixture as compared to heptanal alone [58]. Mating affected these interactions, as OG-PNs in mated males responded with a lower spike frequency when the pheromone was added to heptanal and thus showed suppressive mixture interaction [60]. No effects of pheromone on heptanal responses were recorded electrophysiologically in ORNs and at the AL input using calcium imaging; neither in mated nor in virgin males [15]. Finally, results concerning the processing of mixtures of pheromone and plant odors in the OG also differ among species. In *B. mori* there is no evidence for mixture interactions in OGs [59], whereas in *C. pomonella* suppression was observed in PNs when adding codlemone to non-pheromonal compounds (*i.e*., pear ester, acetic acid and alpha-farnesene) [57].

Various interactions between pheromone and plant odor information occur in the AL, both in the MGC and OG sub-systems, but probably through different mechanisms. The processing of mixtures of sex-pheromone and plant odors depends in some cases on the males’ mating status and varies between species. Pheromone and plant odor information reach different areas of the AL via separate labeled lines, but some ORNs already transmit integrated mixture information. The precise mechanisms of central integration of pheromone and plant odor information are so far unknown. Although some interactions already take place in the peripheral olfactory system, LNs within the AL may contribute to the more complex interactions compared to the peripheral system. Being released by very different sources, pheromones and plant odors probably reach the olfactory organs with fluctuating temporal patterns, very different from the way regular mixtures, *i.e.*, compounds released simultaneously from the same source, are detected, which further brings complexity into the processing of the resulting sensory input in the AL. Further work needs to be done to understand how the local AL network integrates and modulates incoming mixture information to lead to the observed PN responses according to the species, sub-system or mating status.

Although nothing is known on the combinatory integration of sex pheromone and plant odors in higher brain centers, the well-documented involvement of the protocerebrum in multimodal signal integration and learning makes it a good candidate for higher order plant odor-pheromone integration. Olfactory information is transmitted to higher integration centers within the protocerebrum, essentially the lateral protocerebrum (LP) and the calyces of the mushroom bodies (MBs), where projection neurons synapse onto higher order neurons. Olfactory information is essentially integrated with other sensory modalities within the protocerebrum, not excluding some interactions at earlier processing levels, and highly integrated information is finally transmitted to descending pre-motor pathways. Evidence for multimodal integration in the mushroom bodies has been found so far in Hymenoptera and Lepidoptera, where odors and visual signals have been shown to interact (e.g., [61,62]). It is thus likely that multiple cues from the complex sensory environment will converge with information on a behaviorally highly relevant olfactory input such as the sex pheromone at this level. The insect mushroom bodies have also been shown to play an important role in learning and memory. If experience with sensory stimuli modulates responses to the same or other signals, the mushroom bodies might be involved in integrating uni- and multimodal information not only simultaneously, but also scattered in time.

Data obtained in the fruit fly *D. melanogaster* indicate that information collected by third order neurons in the lateral protocerebrum is still segregated as a function of corresponding glomeruli within the AL, whereas Kenyon cells, third order neurons within the mushroom bodies, seem to integrate a wide range of odorants across glomeruli [63,64]. In the tobacco sphinx moth *Manduca sexta*, different projection areas in the lateral protocerebrum have been described even earlier [65] for AL neurons responding either to pheromone or plant odors. Subregions in the inferior lateral protocerebrum were identified depending on projections from AL-PNs originating from different compartments of the macroglomerular complex in the silk moth [66]. On the other hand, Kenyon cell responses in the honey bee have been shown to be highly odor specific [67]. Also in the honey bee, multiglomerular AL-PNs have been shown to project to a distinct area within the lateral protocerebrum, which could potentially be dedicated to odor mixture integration [68]. These findings indicate that elemental mixture processing might dominate in the lateral protocerebrum, preserving highly odor-specific neuronal responses, even though specific regions might still serve for mixture integration. In the mushroom bodies, configural odor mixture processing might be important in some, but not all model insects.

Recordings from different moth species have revealed blend specific higher order olfactory neurons, not responding to individual pheromone components, indicating configural processing of pheromone blends ([47] and references therein). For example, neurons within the lateral accessory lobe of the protocerebrum responded with long lasting excitation only to behaviorally active pheromone blends, but not to single components. It is noteworthy that in *Bombyx mori*, a species that shows orientation behavior to the major pheromone component, bombykol, alone, protocerebral neurons responded to bombykol as a single stimulus ([47] and references therein).

## 4. Plasticity of Pheromone-Plant Odor Interactions

Behavioral responses to specific olfactory signals, such as the sex pheromone, are highly dependent on various external and internal factors. They depend on the sensory environment, which includes not only olfactory cues as plant volatiles, but also stimuli of other modalities, such as gustatory, visual or auditory signals. Presence of such external factors might occur simultaneously with the specific signal or scattered in time and can influence behavioral output through previous experience. On the other hand, internal factors, such as the physiological state of an insect will also modulate responses to specific signals within their sensory context. We will thus now summarize what is known about the plasticity of sex pheromone—plant odor interactions throughout the olfactory system.

It has been shown that mating profoundly changes the physiology in both male and female moths and leads to behavioral modifications in response to olfactory stimuli. Newly mated males transiently stop responding behaviorally to the female-emitted sex pheromone [69,70]. The inhibition of sex pheromone attraction is correlated with a significant increase of the response threshold of pheromone-specific AL neurons in *A. ipsilon*, whereas responses to flower odors in ordinary glomeruli improve after mating [71]. In male *S. littoralis*, post-mating inhibition of behavioral responses to sex pheromone and to the host plant cotton seems to also be correlated with a decrease in peripheral sensitivity, whereas responses to flower odors remain constant after mating [72]. It is not known if *S. littoralis* males change their behavior in the presence of a mixture of the sex pheromone and plant odors, but mating modulates such mixture responses in *A. ipsilon*: virgin males respond better to a mixture of the sex pheromone with a linden flower extract than to the pheromone alone, whereas newly mated males still respond to the flower extract with small amounts of sex pheromone added, but stop responding to the mixture when higher amounts of sex pheromone are applied. Interestingly, only doses of pheromone, which elicited significant firing responses from AL neurons, were able to inhibit upwind flight to floral odor in the wind tunnel [60]. Optical imaging and electrophysiological experiments have shown that, after mating, interactions between sex pheromone and the flower odor remain the same in both sex pheromone specific and flower odor specific ORNs, as well as within the macroglomerular complex of the AL [15]. Neurons within the ordinary glomeruli, on the other hand, respond synergistically to a mixture of sex pheromone and flower odor in virgin males, but flower odor responses are reduced in mated males when the sex pheromone is added [60].

Evidence has accumulated that experience with a sensory stimulus can modulate subsequent responses not only to the same but also to other sensory signals. In this case we can postulate some “delayed interaction” between different sensory signals. Experience with either the sex pheromone or plant odors might modify responses to the other stimulus at a later moment in time. The most striking example is an increased behavioral response to the sex pheromone in *S. littoralis* 24 h after a brief exposure not only to the sex pheromone itself, but also to plant volatiles such as linalool or geraniol. The behavioral response to the sex pheromone even increases after a brief pre-exposure with other sensory modalities such as gustatory or acoustic stimuli (predator sound) [73,74]. This increase in behavioral responses is paralleled by decreased response thresholds of AL neurons within the pheromone processing MGC and in plant odor processing ordinary glomeruli for pre-exposure with the sex pheromone itself or pre-exposure with a non-olfactory sensory signal, a predator sound [73,74]. These cross-modal pre-exposure effects have been postulated to result from a sensory experience-driven maturation of the olfactory system [73]. A similar phenomenon has been described in the codling moth *C. pomonella*. Both males and females pre-exposed to the sex pheromone respond better behaviorally to the kairomonal attractant pear ester [75]. Also, olfactory experience at early stages of a moth’s life can affect adult responses. Larval experience with host plants modulates subsequent reproductive behavior of male moths with consequences on plant-pheromone interactions. *S. littoralis* males reared on a specific host plant as larvae, are, once adults, more attracted to the female sex pheromone in combination with volatiles from the experienced host plant when compared to pheromone and volatiles from a host plant they had not experienced [76].

## 5. Orientation Behavior

Pheromone perception triggers a sustained upwind flight in male moths (positive anemotaxis). In order to follow an intermittent pheromone plume in a turbulent environment, males alternate phases of upwind surge when perceiving pheromone filaments and lateral casting when loosing them (summarized in [1]). Besides the pheromone signal, males rely on mechanical stimuli for the general direction and on visual cues for the control of altitude and ground speed of their flight. We now discuss how a background of general odorants might modify male orientation towards the sex pheromone.

The influence of host plant odors on pheromone-triggered behavior has been studied either under field conditions by comparing the attractiveness of mixed pheromone plant-odor lures to pure pheromone lures, or in a wind tunnel by analyzing the flight behavior of male moths. Under field conditions, adding some of the compounds identified in the volatile emissions of adult or larval food plants to the sex pheromone led to increased catches in pheromone traps in several moth species (see for example: [77,78,79,80]). Mixtures of floral volatiles, including for instance aromatic compounds such as 2-phenylethanol or terpenoids like linalool, used in pure floral baits can often attract not only males but also females [81]. When mixed with the pheromone, they increase the catches of males. Larval-host volatiles, including some green leaf volatiles that are typically attractive to mated females as signals for suitable oviposition sites, also increase pheromone attractiveness [79,81]. In turn, the key floral odorant phenyl-acetaldehyde reduces the number of moths captured compared to pheromone-baited traps [82,83]. Apart from these latter exceptions, the predominant additive or synergistic interactions have been interpreted as an adaptive response of males, increasing the males’ probability to find a female positioned nearby or on an oviposition site or an adult food source. In the field, male moths are attracted to plant volatiles that can guide them towards host trees well before the onset of female pheromone release [84].

Besides those field studies that consider only the scores of male catches, but cannot evidence changes in their navigational behavior, dedicated wind tunnel studies are necessary to understand the mechanisms by which plant odor and pheromone information are integrated. Attraction of male codling moths, *C. pomonella*, to the main pheromone component, codlemone, was greatly enhanced by addition of either component of apple leave emission, (E)-β-farnesene, linalool, or (Z)-3-hexenol in a wind tunnel [85]. In contrast to pheromone blends, the component ratios were not as critical when mixing volatile plant compounds with pheromone. Clear synergistic effects of a blend of larval host volatiles were also observed in the wind tunnel in *Grapholita molesta* [86]. A five-component blend added to the pheromone elicited the highest rate of landings at the odor source in the shortest time, meaning that males flew faster or straighter. In male grapevine moths, *Lobesia botrana*, host plant volatiles added to a sub-optimal pheromone dose (10 times lower than the optimal dose) were also shown to increase the proportions of take off and source contacts up to the response levels of the optimal pheromone dose alone [87]. Interestingly, detailed dose-response experiments showed an optimal ratio (sex pheromone: plant volatiles) of 1:1000. Generally, in all the species investigated so far, doses of plant volatiles largely exceeding those of the pheromone are needed to produce agonistic effects on pheromone-guided behavior. Recorded flight tracks in response to blends of pheromone and plant volatiles in *Eupoecilia ambiguella* revealed that males were activated sooner and reached the source faster in presence of mixed stimuli [88]. Interestingly, synergistic effects of host plant odors were observed not only at under-dosed, as expected in case of additive interactions, but also at overdosed synthetic pheromone blend concentrations. While males stopped in the middle of the wind tunnel at high doses of pheromone, they performed a complete flight to the source in presence of mixtures. This suggests that males could use volatile plant compounds whenever pheromone information is sub-optimal.

**Figure 5:**
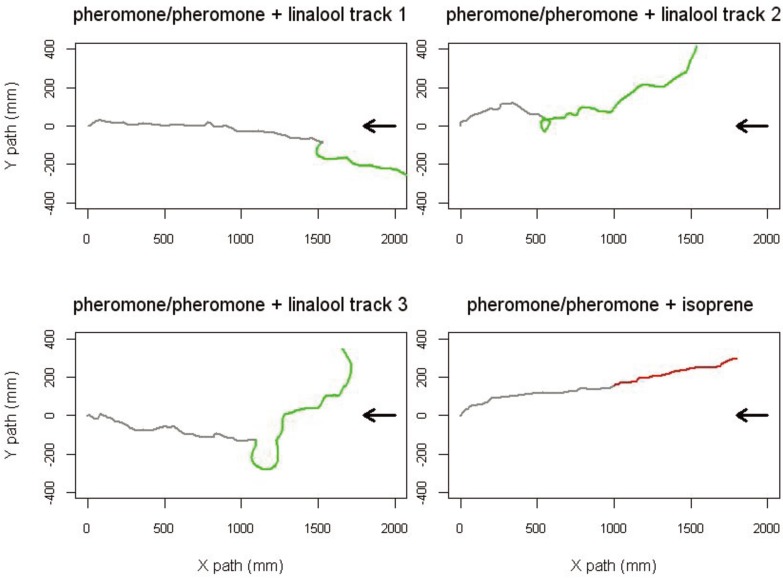
A change of odorant background modifies the orientation behavior of *Spodoptera littoralis* males towards their pheromone. Samples of 2-dimensional walking tracks were recorded with a locomotion compensator. Individual males were stimulated with the main pheromone component, Z9E11-14:Ac, for 2 min and their walking track was recorded showing active upwind walking activity (grey tracks). After 1 min, a plant volatile compound (linalool) was added to the pheromone carrying airflow (green track). Males showed a transitory change in their direction and speed at linalool onset (three examples). In turn, isoprene (red track) did not modify their walking activity. The black arrows indicate the direction of the airflow. Data are redrawn from [14].

Besides these direct effects of the odor environment, insects are expected to encounter very different odorant backgrounds during their flight toward a pheromone source. Sudden changes in background temporarily alter the orientation behavior of male *S. littoralis* walking in a locomotion compensator [14] (Figure 5). When stimulated by an airflow odorized with the main pheromone component, males temporally changed their walking direction at linalool onset, then resumed proper orientation in the pheromone and linalool background. This suggests that a sudden change in the olfactory environment might act as a distractive stimulus to males.

## 6. Adaptive Value of Pheromone-Plant Volatile Interactions

Pheromone communication is very common in insects. Pheromones serve a number of functions in intra-specific communication, and insects living in close contact to plants, such as herbivores, or insects preying on herbivores, are also very sensitive to plant volatile emissions. Thus, with respect to the high diversity of pheromone structure and functions it is difficult to speak of “pheromone-plant odor interactions” in general. However, it is interesting to compare what kind of interactions can be found outside the well-described model of Lepidoptera sex-pheromones to determine whether at least analogous traits might explain why such interactions might have been selected for. Well-documented cases of interactions between pheromones and plant odors are known in Homoptera and a variety of Coleoptera families (Table 1). In these insects, pheromones are involved in attraction of the other sex (sex pheromone) or of both sexes (aggregation pheromones mediating reproductive or feeding behavior). However, pheromone-plant odor interactions also probably occur in many other insect groups. The reported cases of interactions concern pheromones emitted by both females and males (Table 1). A variety of plant-emitted chemicals affect pheromone responses, such as green leaf volatiles, terpenoids, or aromatic compounds, without structural similarity to the respective pheromones. Biologically, such interactions might facilitate mate finding, host exploitation, and even influence specific mate choice.

**Table 1:** Some reported cases of pheromone-plant odor interactions in non-lepidopteran insects.

Insect group	species	Pheromone function	Plant odour type	Compounds	Effects	References
Homoptera (various Aphids)	*Rhopalosiphon padi, Phorodon humuli*	Sex pheromone (female-emitted)	Single host plant volatiles	Benzaldehyde, methyl salicylate	Increased catches to pheromone baited traps in field studies and increased specificity.	[89]
	*Dysaphis plantaginea*		Induced host plant volatiles	Adult-feeding induced green leaf volatiles. (Short chain esters)		[90]
Coleoptera	*Leptinotarsa decemlineata*	Aggregation pheromone (male produced)	Blend of host plant volatiles	(*Z*)-3-hexenyl acetate, linalool, methyl salicylate, nonanal, 2-phenylethanol	Increased attraction in a sex-dependent manner	[91]
	*Melolontha hippocastani, Melolontha melolontha*	Sex pheromone (female emitted)	Induced host plant volatiles	(*Z*)3-hexenal, (*Z*)-2-hexenal, (*E*)-2-hexenal, (*Z*)-3-hexen-1-ol, and other green leaf alcohols	Synergistic attraction of males	[92,93]
	*Pityogenes bidentatus* (Scolytidae)	Aggregation pheromone involved in host colonization	Volatiles from non-host trees	Monoterpenes (α- and β-pinene, terpinolene, 3-carene) and green leaf alcohols, (*Z*)-3-hexenol, (*E*)-2-hexenol, 1-hexanol	Inhibition of attraction to pheromone	[4,94]
	*Rhynchophorus* spp.	Aggregation pheromone involved in reproduction	Natural material or volatiles from damaged host-plant	Short chain products of fermentation (ethyl acetate, acetoin)	Synergy	[95,96,97]

### 6.1. Improving Mate Finding?

In several species belonging to different insect orders (Homoptera, Lepidoptera, Coleoptera), the searching sex is found to respond more to a mixture of pheromone and plant compounds, compared to the pheromone alone. This phenomenon can be linked to habitat localization, during which an insect uses general cue characteristics of the habitat of its host (herbivore or parasite) or prey (predator) to locate the host habitat, and subsequently relies on more specific cues to finally find its host or prey inside the habitat. In oligophagous species, the probability of finding a mate on the host plant is especially high since host plant odors can stimulate pheromone emission (*Homeosoma* moths [98,99]) and even pheromone production (*Heliothis* moths [100]). In less specialized phytophagous moth species, an active choice of females for their calling sites is not documented, and mating can even occur in an environment different from the larval host plant. For these latter species, the adaptive value of synergistic pheromone-host plant interactions remains unclear. In other cases, avoidance of non-host plants whose volatile emissions have been shown to inhibit responses to pheromones could also facilitate mate finding by preventing males from searching within non-suitable habitats [101]. In extreme cases, like in some butterflies where females do not produce any long-range sex attractant, plant volatiles can even constitute the main chemical mate searching cues. Thus, *Heliconius* sp. males locate nearly emerging female pupae by volatile emission of *Passiflora* sp. on which caterpillars have fed and perform mate guarding until emergence of virgin females [102].

### 6.2. Better Host Exploitation?

Aggregation pheromones may be produced by one sex only but attract both sexes. The resulting aggregation behavior unites males and females ready to mate, and apart from serving reproduction, is often associated with feeding on a host plant. In these cases, reproduction and feeding behaviors are intimately associated, and pheromone and plant odors are involved at the same time and place so that their interactions contribute equally to reproduction and host exploitation. Palm weevils for example mate on palm trees on which adults also feed, and partner finding is mediated by a male-emitted pheromone and short chain products of fermentation released by the host tree [95,96,97]. In bark beetles, host-tree emissions attract pioneer individuals, which, once established on a tree, release aggregation pheromones that recruit high numbers of followers. On the other hand, non-host volatiles, for instance green leaf volatiles for bark beetles specialized on coniferous trees, inhibit responses to the aggregation pheromone. Finally, joint action of pheromone and plant odors results in a massive attack of trees enabling bark beetles to overcome the trees’ natural defenses [103].

### 6.3. Better Identification?

In most moths, the chemical composition of the pheromone blends guarantees the species-specificity of attraction; it is the main factor contributing to the reproductive isolation, and the host plant is not necessarily required for mate finding. However, some examples are known in Aphids (Table 1) and tortricid moths, where the pheromone components are shared by different taxa with different host preferences, and males respond more specifically to the pheromone associated to their specific host odor compared to the pheromone alone. Thus, females of the rosy apple aphid, *Dysaphis plantaginea*, emit a 1:8 blend of nepetalactolone and nepatalactol that attracts males. These compounds are shared with several other aphid species and the ratio is not sufficient to guarantee species specificity in mate location. Female aphid infestation induces increased release of four esters from the host leaves. Combination of these esters applied in a 1:1:1 ratio with the pheromone blend increases the number of *D. plantaginea* but decreased the number of other aphids caught in traps [90]. Host races in the larch budworm *Zeiraphera diniana* feed either on larch or on cembran pine*.* Pheromone composition differs significantly between the two host races but cross-attraction can occur at a rate of 0.03–0.38. Cross attraction to larch females increases when they call from neighborhoods rich in pine or on pine trees. Cross-attraction to pine females similarly increases when calling from neighborhoods rich in larch. The plant environment thus affects assortative attraction to pheromone [104,105,106,107]. These cases are particularly interesting since they provide model systems to address the role of host adaptation in assortative mating and sympatric speciation.

## 7. Conclusions

The research summarized in this review shows that insects use their surrounding odor landscape in manifold ways when responding to pheromones. On the other hand, abundant volatiles can also mask crucial odors and challenge the chemical specificity of specialized receptors potentially imposing selective pressure on insects to develop neuronal mechanisms to extract relevant information from an odor background. The biological relevance of either outcome of interactions between pheromone and plant odor information is still under discussion and most likely depends on the insect species, the environmental context and the precise compounds involved.

Pheromone-plant odor interactions might also play a role in the evolution of mating communication systems and their contribution to reproductive isolation. There are a few examples in the literature showing that the attraction specificity of reconstituted pheromone blends depends on the plant on which the traps have been settled [108]. Host plant preferences may reinforce assortative mating controlled by the composition of the pheromone blend [104,105,106]. The formation of host races of moths for example with a co-evolution of female preference for certain hosts and male preferences for pheromone blends emitted by females associated with a specific host passes most likely via complex sensory input of both types of odors. In agro-ecosystems, newly appearing interactions of different types of odors might contribute, for example, to the success of newly introduced invasive species. On the other hand, detailed knowledge of interaction mechanisms might help to develop new integrated pest management strategies by profiting for example from the masking of odors important for reproduction by plant-derived volatiles.

So far, most studies on odor interactions treat behavioral and neuronal responses during simultaneous stimulation, however, recent publications [13,14] aim at mimicking a more natural situation and show that it is important to take spatial and temporal aspects of stimulation into consideration when analyzing responses to pheromone-plant odor mixtures. From a mechanistic point of view, we are still far from understanding the whole process of pheromone-plant odor interactions. Here, we gather evidence that different odors already interact at the detection level, even in highly specific systems, such as the moth pheromone system, which were long considered to possess highly specific receptor neurons and function along labeled lines throughout the first levels of the olfactory pathway. Which part the subsequent integration levels play in such interactions needs to be further dissected in the future. We postulate that convergence of different ORNs and the network of local neurons within the AL contribute to mixture response patterns in AL output neurons, which exhibit more complex interaction features than individual ORNs. How the observed interactions within the brain are subsequently translated into motor output patterns and behavior is still to be unraveled. Recent literature shows in addition that pheromone-plant odor interactions are not static through hard-wired pathways, but submitted to modulation, allowing adaptive behavior as a function of internal factors such as physiological state and external factors e.g., experience.
